# Lack of telomerase reduces cancer incidence and increases lifespan of zebrafish *tp53*^*M214K*^ mutants

**DOI:** 10.1038/s41598-024-56153-8

**Published:** 2024-03-05

**Authors:** Naz Şerifoğlu, Bruno Lopes-Bastos, Miguel Godinho Ferreira

**Affiliations:** Institute for Research on Cancer and Aging of Nice (IRCAN), UMR7284, INSERM U1081, CNRS, Université Cote d’Azur, 06107 Nice, France

**Keywords:** Telomerase, p53, Aging, Cancer, Zebrafish, Cancer, Ageing

## Abstract

Telomerase activity is restricted in humans and telomere attrition occurs in several tissues accompanying natural aging. Critically short telomeres trigger DNA damage responses and activate p53 which leads to apoptosis or replicative senescence. These processes reduce cell proliferation and disrupt tissue homeostasis, thus contributing to systemic aging. Similarly, zebrafish have restricted telomerase expression, and telomeres shorten to critical length during their lifespan. Telomerase-deficient zebrafish (*tert −/−*) is a premature model of aging that anticipates aging phenotypes due to early telomere shortening. *tert −/− *zebrafish have impaired cell proliferation, accumulation of DNA damage markers and p53 response. These cellular defects lead to disruption of tissue homeostasis, resulting in premature infertility, gastrointestinal atrophy, sarcopenia and kyphosis. Such consequences contribute to its premature death. Here we reveal a genetic interdependence between *tp53* and telomerase function. Mutation of *tp53* abrogates premature aging of *tert −/− *zebrafish, prolonging male fertility and lifespan. However, it does not fully rescue healthspan. *tp53mut tert −/− *zebrafish retain high levels of inflammation and increased spontaneous cancer incidence. Conversely, loss of telomerase prolongs the lifespan of *tp53mut* single mutants. Lack of telomerase reduces two-fold the cancer incidence in double mutants and increases lifetime survival. Thus, we observe a reciprocal rescue of *tp53mut* and *tert −/− *that ameliorates lifespan but not spontaneous cancer incidence of *tp53mut*, likely due to higher levels of inflammation.

## Introduction

Eukaryotic chromosome ends are composed of specialized nucleoprotein complexes, known as the telomeres, that play a crucial role in maintaining genome stability and preserving genetic material^1,2^. They are maintained by telomerase, a reverse transcriptase, that adds (TTAGGG)_n_ repeats to chromosome ends^2,3^. In human somatic cells, telomerase activity is restricted and telomeres shorten in most somatic cells with each round of cell division due to the “end-replication problem"^4,5^. Telomere shortening acts as a molecular counting mechanism, reflecting the number of divisions that have occurred, and hence constituting a hallmark of aging^4,5^. Telomeres become dysfunctional due to critical shortening or deprotection^6^. Dysfunctional telomeres are sensed as DNA damage and depending on tissue proliferation demands and p53/p63/p73 status, they either initiate an apoptotic response or G1 cell cycle arrest leading to senescence^7,8^.

Telomere maintenance is crucial for cell survival and replicative potential, and influences tissue homeostasis^9^. Impaired tissue homeostasis lies at the core of age-associated diseases and telomere biology disorders (TBDs)^10^. TBDs are genetic disorders that result from mutations in genes involved in telomere structure and maintenance. Patients with TBDs show accelerated telomere attrition and reduced cell proliferation, display premature onset of aging phenotypes and shorter lifespans^11,12^. For example, in dyskeratosis congenita there is a direct correlation between disease severity and telomere length^13^.

Most of our knowledge on vertebrate telomere biology comes from studies conducted in inbred mice strains that possess long telomeres^14^. Several generations of incrossing of telomerase KO mice are required to attain short telomeres with a noticeable impact at the organism level^15–17^. Data from late generation telomerase KO mice suggest that apoptosis and/or senescence play a crucial role in the development of degenerative phenotypes^18,19^. Deletion of *Puma* (p53 up-regulated modulator of apoptosis) or *Cdkn1a* (p21 CDK inhibitor) separately ameliorate the degenerative phenotypes of late generation telomerase KO mice^19,20^. In *Terc* (Telomerase RNA component) KO mice, stem cell exhaustion mediated by *Puma*-induced apoptosis, is responsible for limiting the organism’s lifespan^20^. The wild-derived Cast/Ei mice have been proposed as a more suitable model for studying telomere dysfunction in humans due to its naturally shorter telomeres^21^. First generation telomerase Cast/Ei KO mice exhibit defects that better resemble human TBDs^21^.

Zebrafish have telomeres with length similar to those of humans. More importantly, their telomeres also undergo critical shortening during their lifetime, which makes them a suitable vertebrate model to study aging and TBDs^22–25^. Specifically, telomerase deficient zebrafish (*tert −/−*) exhibit accelerated telomere shortening leading to reduced cell proliferation, premature tissue damage, early onset of aging phenotypes, and shortened lifespan^22–25^. *tert −/− *zebrafish also display chronic inflammation, increased susceptibility to infections and accelerated incidence of cancer^26,27^.

*TP53* is a tumor suppressor gene that plays a crucial role as the “guardian” of genome. Mutations in *TP53* are found in more than half of all human tumors. *TP53* regulates cell cycle progression and genome stability by activating DNA repair, apoptosis and senescence. Zebrafish *tp53*^*M214K*^ mutants (equivalent to human M246 in the DNA binding domain and mutated in several cancers) lack transcription of p21, *Puma* and *Bax* and G1 checkpoint activation^28^. Similar to mice and humans, *tp53*^*M214K*^ zebrafish mutants develop soft-tissue spontaneous tumors by the age 9 months^28^.

Several phenotypes associated to deficiency of telomerase require p53 function. Deletion of p53 is sufficient to prevent germ-cell apoptosis and infertility in late-generation *Tert −/− *mice^29^. However, this comes at the high cost of increased genome instability and cancer^29,30^. In *tert −/− *zebrafish, although *tp53* deficiency rescues cell-proliferation defects and premature death^12^, its effect at the organism level and tumor incidence remained unknown. Conversely, it was not investigated if lack of telomerase would have an impact on *tp53* mutant zebrafish. Here we show that *tp53* mutation rescues male fertility, attenuates aging phenotypes, and increases lifespan of *tert −/− *zebrafish. However, similar to *tert −/− *fish, double mutants retain higher inflammation and early cancer incidence when compared to wild type.

## Results

### *tp53* mutation rescues premature male infertility of *tert −/− *zebrafish

**Figure 1:**
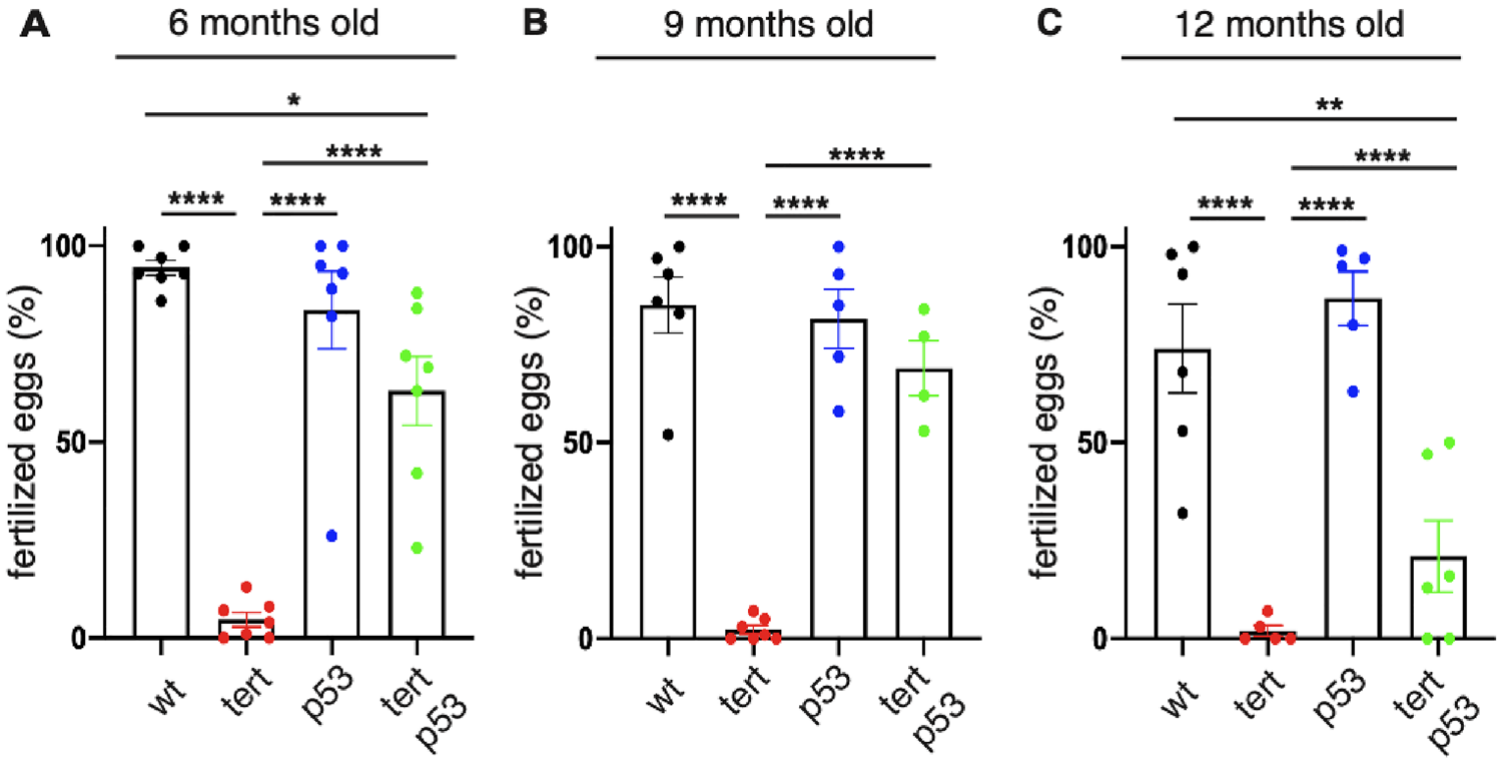
*tp53mut* rescues the premature infertility of the telomerase deficient zebrafish. (**A**) quantification of *tert −/− *male fertility at 6 months of age (n_WT_ = 7, n_*tert−/−*_ = 7, n_*tp53mut*_ = 7, n_*tert−/− tp53mut*_ = 7) (**B**) quantification of *tert −/− *male fertility at 9 months of age (n_WT_ = 6, n_*tert−/−*_ = 7, n_*tp53mut*_ = 5, n_*tert−/− tp53mut*_ = 4 (**C**) quantification of *tert −/− *male fertility at 12 months of age (n_WT_ = 6, n_*tert−/−*_ = 5, n_*tp53mut*_ = 5, n_*tert−/− tp53mut*_ = 6). Each value in the graphic represents the mean of a minimum and maximum number of eggs of 5–7 crosses. Statistical tests were performed using a one-way ANOVA, **** p < 0.0001, ***p < 0.001, **p < 0.01, *p < 0.05.

Loss of male fertility is one of the early phenotypes of zebrafish aging in both wild type and *tert*^*hu3430/hu3430*^ mutants (referred as *tert −/− *in this study)^23^. *tp53*^*M214K/M214K*^ homozygous mutants (*tp53mut* in this study) rescues cell proliferation decline of testis and gut of *tert −/− *zebrafish, resulting in partial restoration of tissue integrity^22^. To test whether *tp53* mutation had a functional effect on *tert −/− *zebrafish fertility, we have conducted fertility assays over their lifespan. We selected 6-month-old fish as the earliest time point, outcrossed mutant males with wild type females and evaluated the percentage of fertilized eggs. Consistent with our previous reports, by the age of 6 months, *tert −/− *males were almost completely infertile (Fig. 1A, WT vs *tert −/− p* < *0.0001, tp53mut* vs *tert −/− *p < 0.0001*, tert −/− *vs *tert −/− ; tp53mut* p < 0.0001). In the *tert −/− ; tp53mut* background, fertility was slightly lower than that of wild type males (Fig. 1A, WT vs *tert −/− ;tp53mut p* = *0.014*). However, approximately 60% of the eggs laid by wild-type females were fertilized, indicating a clear functional rescue of *tert −/− *male infertility (Fig. 1A, *tert −/− *vs *tert −/− ;tp53mut p* < *0.0001*).

Fertility is progressively lost in *tert −/− *males with less than 3% of eggs fertilized by 9 months of age (Fig. 1B, WT vs *tert −/− *p < 0.001). In contrast, 9-month-old *tert −/− ; tp53mut* males maintained their fertility, with approximately 70% of eggs successfully fertilized (Fig. 1B, WT vs *tert −/− ; tp53mut* ns p = *0.283, tert −/− *vs *tert −/− ; tp53mut* p < 0.0001). At the age of 12 months, *tert −/− ; tp53mut* males showed a significantly reduced fertility when compared to wild type and *tp53mut* siblings. (Fig. 1C, WT vs *tert −/− ; tp53mut* p = 0.001, *tp53mut* vs *tert −/− ; tp53mut* p < 0.001). *tert −/− ; tp53mut* male fertility also declined with age with approximately 20% of the eggs laid by wild type females. This difference was not statistically significant from *tert −/− *siblings (Fig. 1C, *tert −/− *vs *tert −/− ; tp53mut* ns p = 0.427). Decline of fertility in double mutants is likely due to the terminal block to cell proliferation imposed by telomere shortening known as Hayflick M2/Crisis as observed in primary human cells lacking p53 function.

### *tp53* mutation reduces *tert −/− *premature aging phenotypes

**Figure 2:**
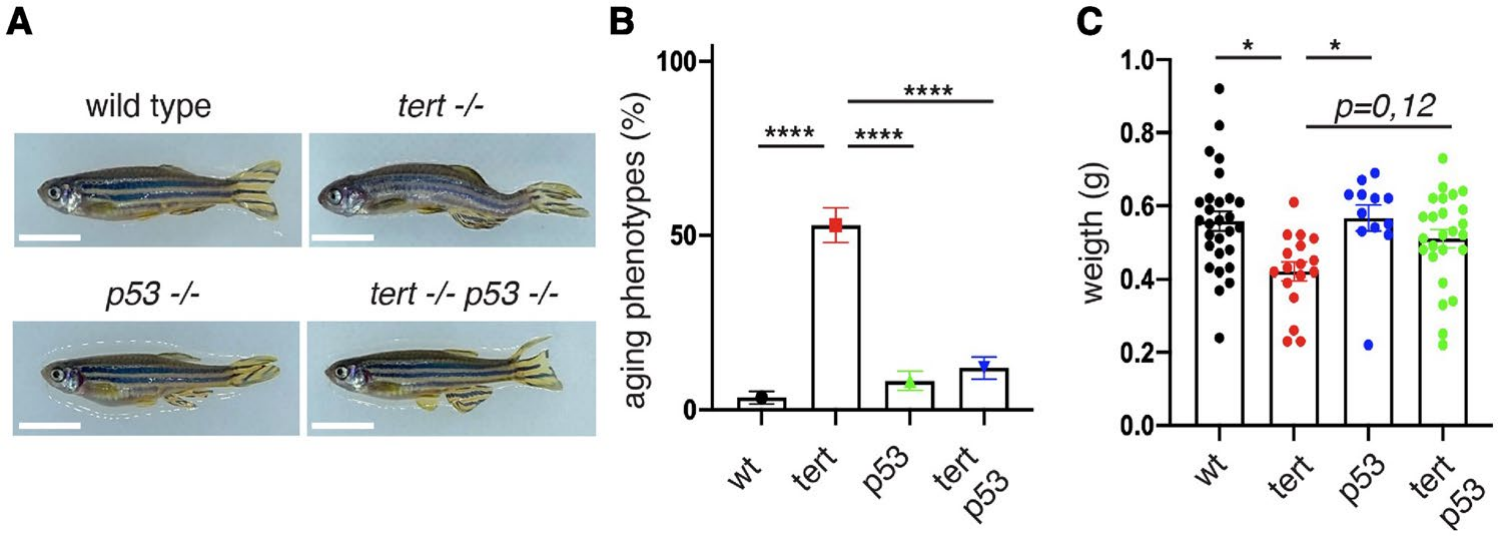
*tp53* rescues early incidence of *tert −/− *cachexia and kyphosis. (**A**) representative images of 12-month-old zebrafish of different genotypes. Bar represents 1 cm. (**B**) quantification of aging phenotypes in adult zebrafish (n_WT_ = 28, n_*tert−/−*_ = 17, n_*tp53mut*_ = 12, n_*tert−/− tp53mut*_ = 25). (**C**) quantification of weight in adult zebrafish (n_WT_ = 28, n_*tert−/−*_ = 17, n_*tp53mut*_ = 12, n_*tert−/− tp53mut*_ = 25). Statistical tests were performed using a one-way ANOVA, **** p < 0.0001, *p < 0.05.

The rescue of male fertility prompted us to investigate whether *tp53* mutation improved other aging phenotypes, such as kyphosis (abnormal curvature of the spine), caused by increased weakness of the spinal bones, and cachexia (excessive muscle wasting), caused by muscle tissue atrophy. The incidence of these phenotypes was monitored from 12 months of age until the time of death. At 12 months of age, 52% of *tert −/− *zebrafish developed cachexia and/or kyphosis (Fig. 2A and B, WT vs *tert −/− *p < 0.001*, tert −/− *vs *tert −/− ;tp53mut* p < 0.001). This increase in incidence of aging phenotypes was accompanied by weight loss of *tert −/− *zebrafish by a mean weight of about 0.4 g compared to 0.55 g in wild type and *tp53mut* siblings (Fig. 2C, WT vs *tert −/− *p = 0.004*, tp53mut* vs *tert −/− *p = 0.016)*.* In the *tert −/− ;tp53mut* background, cachexia and kyphosis incidence was about 13% and significantly lower than *tert −/− *siblings (Fig. 2B, *tert −/− *vs *tert −/− ;tp53mut* p < 0.001*)*.

### *tp53* mutation does not reduce *tert −/− *inflammation

**Figure 3:**
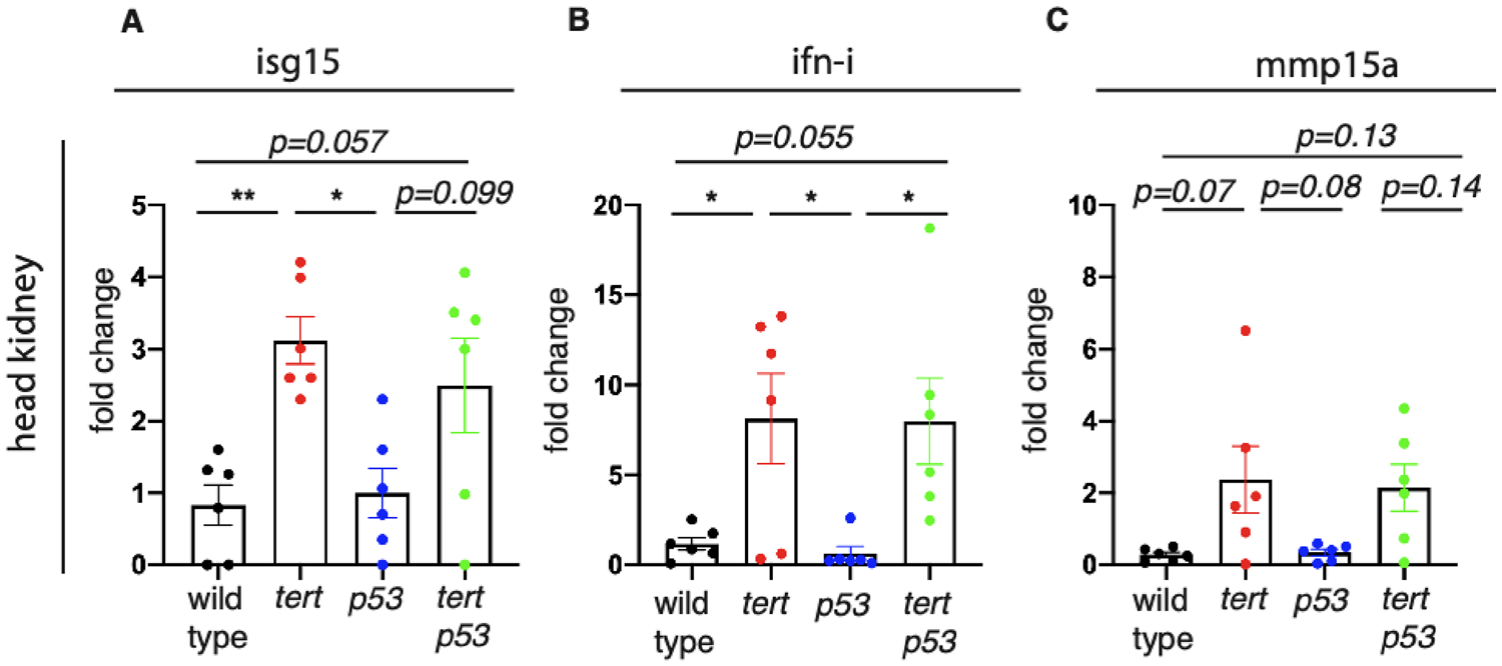
*tp53* does not rescue chronic inflammation of the telomerase-deficient zebrafish. (**A**) RT-qPCR analysis of *isg15* in head kidney (n_WT_ = 6, n_*tert−/−*_ = 6, n_*p53−/−*_ = 6, n_*tert−/− tp53mut*_ = 6). (**B**) RT-qPCR analysis of *ifn-i* in head kidney (n_WT_ = 6, n_*tert−/−*_ = 6, n_*p53−/−*_ = 6, n_*tert−/− tp53mut*_ = 6). **(C**) RT-qPCR analysis of *mmp15a* in head kidney (n_WT_ = 6, n_*tert−/−*_ = 6, n_*p53−/−*_ = 6, n_*tert−/− tp53mut*_ = 6). Statistical tests were performed using a one-way ANOVA, **p < 0.01, *p < 0.05.

Low-grade sterile inflammation is a recognized feature of aging and is a preeminent phenotype of *tert −/− *zebrafish. We have recently shown that senescence-associated inflammatory phenotype (SASP) and inflammation are key features of telomerase mutants that are initiated by specific organs during aging^26^. To test whether *tp53* mutation could attenuate the effects of increased cell senescence of *tert −/− *zebrafish, we examined the expression of genes related to type I interferon inflammation (Interferon-stimulated gene 15, *isg15* and Type I interferon, *ifn-i*)^31^ and SASP (matrix metallopeptidase 15a, *mmp15a*)^32,33^ in 12-month-old zebrafish. The head kidney was chosen because it hosts the adult hematopoietic system and produces blood cells that regulate communication between organs. In *tert −/− *zebrafish, inflammation significantly higher than wild type and *tp53mut* siblings (*isg15,* Fig. 3A, WT vs *tert −/− *p = 0.006, *tp53mut* vs *tert −/− *p = 0.012) and (*ifn-i*, Fig. 3B, WT vs *tert −/− *p = 0.049, *tp53mut* vs *tert −/− *p = 0.031). Similarly, expression levels of the matrix metalloproteinase *mmp15a* associated with inflammation and SASP were elevated in *tert −/− *zebrafish (Fig. 3C). However, inflammation observed in *tert −/− *zebrafish was not reduced in *tert −/− ;tp53mut* (Fig. 3A–C), denoting an incomplete rescue of double mutants likely due to ongoing DNA damage triggered by critically short telomeres.

### *tert −/− *reduces tumor incidence of *tp53mut* zebrafish

**Figure 4:**
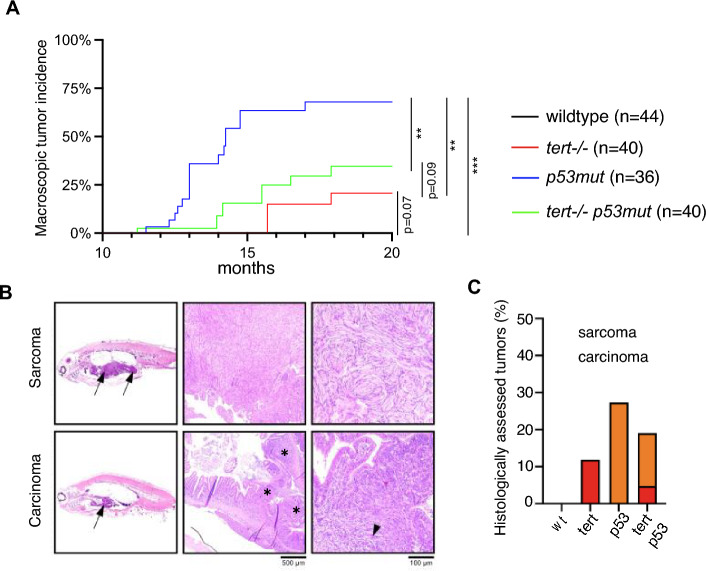
Absence of telomerase reduces earlier and higher tumor incidence of *tp53mut*. (**A**) quantification of spontaneous tumors through macroscopic analysis (n_WT_ = 44, n_*tert−/−*_ = 40, n_*p53−/−*_ = 36, n_*tert−/− tp53mut*_ = 40). Log-rank test was performed to determine statistical differences between the different groups. ***p < 0.001, **p < 0.01. Loss of telomerase also modifies the tumour profile of *tp53mut*. (**B**) Example of haematoxylin and eosin (HE) staining of a soft tissue sarcoma (arrows, upper panel) and an intestinal adenocarcinoma (arrow, lower panel). Sarcoma of *tp53mut* expanded to the abdominal cavity, infiltrating adipose tissue, pancreas, liver and intestine and was histologically consistent with malignant peripheral nerve sheath tumor (MPNST). The carcinoma of *tert −/− *expanded and effaced the intestinal mucosa and submucosa (asterisk), corresponding to a loosely cellular, unencapsulated, poorly circumscribed neoplasm composed of polygonal cells arranged in poorly cohesive cords (black arrowhead). Original magnification, 5x (bar 500 μm) and 20x (bar 100 μm). (**C**) quantification of spontaneous tumors through histological analysis in 12 months old fish (n_WT_ = 25, n_*tert−/−*_ = 59, n_*p53−/−*_ = 22, n_*tert−/− tp53mut*_ = 21). Number of tumors were not significantly different between mutants (Chi-square test).

During our observation of zebrafish aging, we noticed that *tp53mut* zebrafish started to develop macroscopically visible tumors from the age of 11 months on. By the time they reached 18 months of age, about 65% of *tp53mut* fish had developed visible tumors (Fig. 4A, WT vs *tp53mut* p < 0.001). At this time, incidence of cancer was 30% in *tert −/− ;tp53mut* (Fig. 4A, WT vs *tert −/−; tp53mut* p < 0.001) and 15% in *tert −/− *zebrafish (Fig. 4A, WT vs *tert −/− *ns p = 0.072). However, a more detailed histological analysis of 12 months old fish revealed that macroscopic assessment of tumors led to underestimation of true tumor incidence and onset. We were able to detect both carcinomas and sarcomas in these fish (Fig. 4B). Nevertheless, all genotypes exhibited a similar trend concerning susceptibility to tumor incidence. Approximately 10% of *tert −/− *zebrafish developed tumors, exclusively carcinomas (Fig. 4C). In *tert −/−; tp53mut* zebrafish, tumor incidence was about 20%, and most tumors were sarcomas. Consistent with the macroscopic assessment, *tp53mut* had a high tumor incidence, of approximately 30%, all sarcomas. In contrast to 18 months of age, tumor incidence at 12 months did not reach statistical significance between mutant genotypes (Fig. 4C).

### *tert −/− *rescues *tp53mut* reduced lifespan

**Figure 5:**
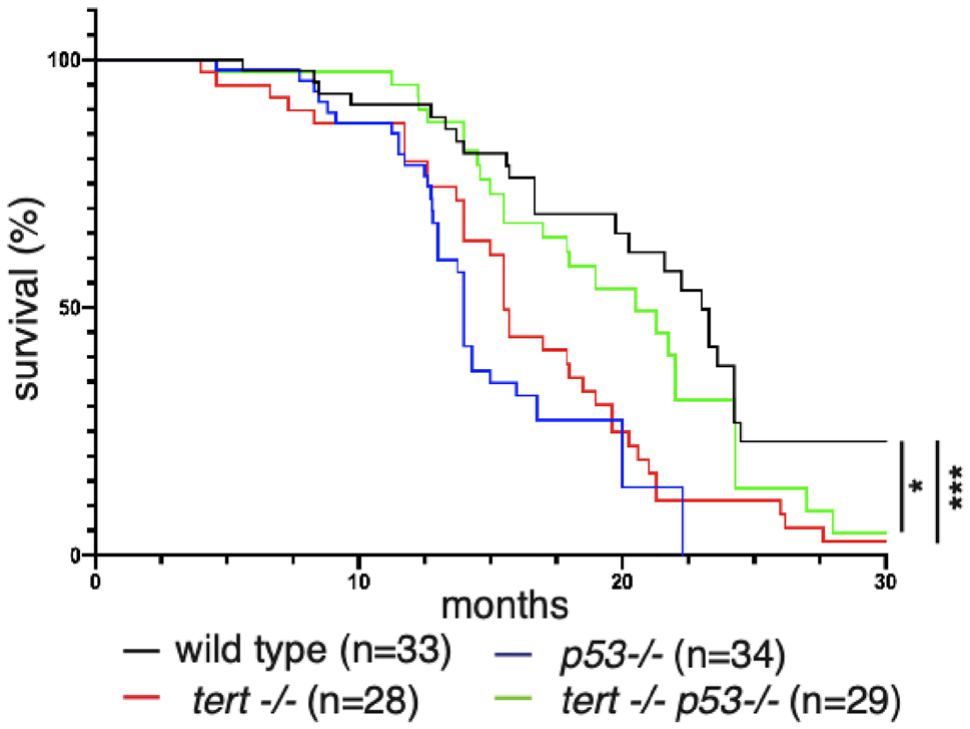
Lack of telomerase rescues the lifespan of *tp53* deficient zebrafish. quantification of survival of zebrafish from 6 to 30 months after birth. Median lifespans *tp53mut:* 14 months, *tert −/−:* 15.5 months, WT and *tert −/− tp53mut:* 20.5 months (n_WT_ = 33, n_*tert−/−*_ = 28, n_*p53−/−*_ = 34, n_*tert−/− tp53mut*_ = 29). Log-rank test was performed to determine statistical differences between the different groups. ***p < 0.001, *p < 0.05.

Telomerase deficiency and dysfunctional telomeres result in reduced lifespan^11–13,15^. Inhibition of p53 via pifithrin-alpha (PFTa) and morpholinos against *tp53* were shown to increase median life span of G2 *tert −/− *zebrafish larvae^24,25^. Consistent with previous work, *tert −/− *zebrafish exhibited a shorter lifespan than wild-type fish with a median of 15.5 months (Fig. 5, WT vs *tert −/− *p < 0.001). Unexpectedly, *tp53mut* fish had the shortest life span, with a median of 14 months (Fig. 5, WT vs *tp53mut* p < 0.001), primarily due to malignant tumor related deaths. Strikingly, *tert −/− ;tp53mut* zebrafish lived as long as wild type fish, with a median lifespan of 20.5 months (Fig. 5, WT vs *tert −/− ;tp53mut* ns p = 0.118, *tert −/− *vs *tert −/− ;tp53mut* p = 0.044, *tp53mut* vs *tert −/− ;tp53mut* p < 0.001). Overall, our data shows that phenotypes associated with telomerase deficiency (premature infertility, cachexia and kyphosis) are rescued by loss of p53 function leading to increased longevity. However, loss of p53 does not rescue high inflammation and anticipated cancer incidence observed in *tert* mutants.

## Discussion

Decline in cell proliferation constitutes one of the most prominent physiological changes that occurs during aging, which ultimately leads to tissue dysfunction^30^. Telomere attrition is a shared characteristic of both normal aging and premature aging syndromes. Zebrafish *tert −/− *mutants exhibit premature short telomeres resulting in increased levels of DNA damage markers, 53BP1 and P-γH2AX^22^. Importantly, DNA damage markers colocalize with telomeres in *tert −/− *zebrafish^23^. DNA damage response was corroborated by increase of p53 levels and transcription of its target genes: *puma*, *cyclinG1* and *cdkn1a*^22^. Proliferative tissues showed a marked decline of cell proliferation (PCNA) with an initial increase in apoptosis (TUNEL) and, later, cell senescence (SA-B-Gal, p15/16 and p21), accompanied by increased mitochondria dysfunction (disrupted mitochondria membranes, low ATP and increased ROS) and appreciated tissue damage^22,23,25^. Our current study extends the understanding of the *tp53-*dependent effects of telomere shortening at the organism level and how p53 stabilization accelerates aging phenotypes. Loss of *tp53* function in telomerase mutants extends the fertile period of males and, at the organism level, ameliorates aging phenotypes and longevity. These findings provide further support to the hypothesis that aging is a consequence of loss of tissue homeostasis partly due to reduced cell proliferation.

Inhibition of p53 increases longevity of *tert −/− *zebrafish, but the underlying mechanism is not unidirectional. We previously reported that *tert −/− *zebrafish are more susceptible to tumorigenesis and develop spontaneous tumors, mostly carcinomas, at an earlier age^23^. Loss of p53 activity is also strongly associated with increased cancer incidence^34^. Approximately 28% of *tp53mut* zebrafish develop sarcomas by 9 months of age^28^. Consistent with these findings, we observed an acceleration in spontaneous tumor formation upon loss of *tp53*. Interestingly, *tert −/− *developed spontaneous tumors later and less frequently than *tp53mut* zebrafish, suggesting that while telomerase deficiency drives tumorigenesis, it can also restrain the onset of *tp53mut* tumors. Lack of telomerase also modifies the types of tumors of *tp53mut,* as double mutants now exhibited not only sarcomas but a majority of carcinomas.

While scoring for tumor incidence, we observed that *tp53mut* zebrafish died shortly after developing tumors. Survival analysis revealed that among the four genotypes, *tp53mut* zebrafish had the shortest lifespan. Importantly, the shortened lifespan observed in both *tp53mut* and *tert −/− *was rescued when both genes were mutated. These findings are in agreement with previous studies that used p53 morpholinos and pharmacology-based methods to inhibit p53 in G2 *tert −/− *larvae^24,25^. However, these studies did not explore the effect of p53 mutations on lifespan, suggesting a unidirectional rescue, whereas we now show a mutual rescue between *tp53mut* and *tert −/− *zebrafish.

In addition to proliferative arrest, chronic inflammation is another consequence of telomere shortening that contributes to the fragility of *tert −/− *zebrafish and accelerated tumor incidence. The source and mechanism of action of short telomere-driven inflammation is not fully identified yet. Dead, damaged, or stressed tissues can trigger chronic inflammation^35^. In our previous studies, we showed that tissue integrity was improved in the *tert −/− ;tp53mut* zebrafish. However, these improvements did not rescue the inflammation signature in the kidney marrow, the zebrafish hematopoietic organ. Our work reveals that restoring cell proliferation and improving tissue integrity rescues several premature aging phenotypes of *tert −/− *zebrafish, such as premature infertility, body wasting and reduced life span. However, like in our previous studies^27^, chronic inflammation is not rescued and allowing cell proliferation in presence of persistent DNA damage have pro-tumorigenic effects, ultimately impairing the healthspan of the organism.

## Methods

### Ethics statement

All zebrafish studies and methods were performed in accordance with the guidelines and regulations of the IRCAN Animal Care Committee, in addition experimental protocols were approved by the regional (CIEPAL Cote d’Azur #784) and national authorities (French Ministry of Research #27673-2020092817202619). This study was conducted as recommended by the ARRIVE guidelines.

### Zebrafish lines and maintenance

Zebrafish were maintained in accordance with Institutional and National animal care protocols. The stock line was preserved as *tert*^*hu340/*+^ *tp53*^*M214K/*+^ double heterozygote and maintained strictly by outcrossing it to AB WT fish to avoid the effects of haploinsufficiency in the progeny. Experimental fish were obtained by in-crossing the stock line. Overall characterization of these four genotypes was performed in F1 sibling animals at 9 months of age. Due to male sex bias in our crosses, that affected mostly *tert −/− *progeny, we were unable to obtain significant numbers of females for analysis and so all data are restricted to males, except the survival analysis, scoring of aging phenotypes and macroscopic tumor formation.

### Fertility assays

In order to assess male fertility, single 9-month-old males from the four different genotypes were separately housed overnight in external breeding tanks with a single young (3–6 months old) wild type female. Breeding pairs were left to cross and lay eggs in the following morning and embryos were collected approximately 4 h post fertilization (hpf) and allowed to develop at 28 °C. Assessment of fertilized eggs and embryo viability was conducted between 4 and 6 hpf. At least 12 independent crosses were conducted for each genotype to evaluate male fertility. Only successful breeding trials, defined as events in which a clutch of eggs was laid by the female, were scored.

### Real-time quantitative PCR

Twelve-months-old zebrafish were sacrificed in 1 g/L of MS-222 (Sigma Aldrich) and the head kidney was collected and immediately snap frozen in liquid nitrogen. RNA extraction was performed in TRIzol (Invitrogen) by mashing tissues with a motorized pestle in a 1.5 mL eppendorf tube. After incubation at room temperature (RT) for 10 min TRIzol, chloroform extractions were performed. Quality of RNA samples was assessed through BioAnalyzer (Agilent 2100). Retro-transcription into cDNA was performed using QuantiTect Reverse Transcription kit (Qiagen).

Quantitative PCR (qPCR) using primers described in Table 1 was performed using FastStart Universal SYBR Green Master mix (Roche) and an StepOne plus Real time PCR Detection System (Applied Biosystems). qPCRs were carried out in duplicate for each cDNA sample. Relative mRNA expression was normalized against the housekeeping gene Ribosomal protein S11 (*rps11*) mRNA expression using the 2^−ddCT^ method compared to control condition.

**Table 1:** Primers used for RTqPCR.

	Gene ID	Forward primer	Reverse primer
*isg15*	ZDB-GENE-021211-1	ACTCGGTGACGATGCAGC	TGGGCACGTTGAAGTACTGA
*ifn-i*	ZDB-GENE-030721-3	CAAGATACGCAAAGCCAGCA	GTGGCTTTTCACAACTCTCC
*mmp15a*	ZDB-GENE-070817-4	GGGTCATGCTCTGGGGGTTGG	AGTGGTGACAGTCTCTGGAGATCCA
*rps11*	ZDB-GENE-040426-2701	ACAGAAATGCCCCTTCACTG	GCCTCTTCTCAAAACGGTTG

### Scoring of aging phenotypes

Aging fish were observed macroscopically for signs of body wasting (cachexia) by evaluating the dorsal–ventral width at the dorsal fin level and comparing with their length, as performed in our previous study^22^. To further evaluate body wasting, fish were weight at the same age. Spinal curvature was assessed macroscopically as any age-associated deformation of the spine in adult fish.

#### Macroscopic assessment of tumors

Fish were screened weekly for the presence of macroscopic tumors as Berghmans et al.^28^. Through this assessment, we could identify the presence of macroscopic tumors in the abdominal and flank region, eye, and gills.

### Fixation for histology and tumor evaluation

Twelve months old zebrafish were euthanized with 1 g/L of MS-222 (Sigma, MO, USA), followed by fixation in 10% neutral buffered formalin for 72 h and decalcified in 0.5 M EDTA for 48 h at room temperature. Samples were then paraffin-embedded to perform 5 mm sagittal section slides. Slides were stained with haematoxylin and eosin and assessed for the presence of tumors by a histopathologist. For this analysis, tumors were grouped in sarcomas, which develop in the connective tissue, such as vessels, nerve bones, muscle and cartilage, and carcinomas which develop in the epithelial tissue.

### Statistical analysis

Graphs and statistical analyses were performed in GraphPad Prism8 software, using one-way ANOVA test Tukey’s post-correction. A critical value for significance of p > 0.05 was used throughout the study. For survival analysis, Log-rank tests were performed using GraphPad Prism8 to determine statistical differences of survival curves.

## Data Availability

All data generated or analysed during this study are included in this published article.
